# Author Correction: A VEGF receptor vaccine demonstrates preliminary efficacy in neurofibromatosis type 2

**DOI:** 10.1038/s41467-020-16007-z

**Published:** 2020-04-21

**Authors:** Ryota Tamura, Masato Fujioka, Yukina Morimoto, Kentaro Ohara, Kenzo Kosugi, Yumiko Oishi, Mizuto Sato, Ryo Ueda, Hirokazu Fujiwara, Tetsuro Hikichi, Shinobu Noji, Naoki Oishi, Kaoru Ogawa, Yutaka Kawakami, Takayuki Ohira, Kazunari Yoshida, Masahiro Toda

**Affiliations:** 1 Department of Neurosurgery, Keio University School of Medicine, 35 Shinanomachi, Shinjuku-ku, Tokyo, 160-8582 Japan; 2 Department of Otorhinolaryngology, Head and Neck Surgery, Keio University School of Medicine, 35 Shinanomachi, Shinjuku-ku, Tokyo, 160-8582 Japan; 3 Department of Pathology, Keio University School of Medicine, 35 Shinanomachi, Shinjuku-ku, Tokyo, 160-8582 Japan; 4 Department of Radiology, Keio University School of Medicine, 35 Shinanomachi, Shinjuku-ku, Tokyo, 160-8582 Japan; 5 OncoTherapy Science, Inc., 3-2-1, Sakado, Takatsu-ku, Kawasaki City, Kanagawa 213-0012 Japan; 6 Division of Cellular Signaling Institute for Advanced Medical Research, Keio University School of Medicine, 35 Shinanomachi, Shinjuku-ku, Tokyo, 160-8582 Japan

**Keywords:** Immunology, Medical research, Neurology, Cancer immunotherapy, Tumour vaccines

Correction to: *Nature Communications* 10.1038/s41467-019-13640-1, published online 17 December 2019.

The original version of this Article omitted from the author list the 10th author Tetsuro Hikichi, who is from OncoTherapy Science, Inc., 3-2-1, Sakado, Takatsu-ku, Kawasaki City, Kanagawa, 213-0012, Japan. This author has been added to the article. Consequently, the 5th sentence in the Authors Contributions has been modified to read “RT, MF, YM, KO, KK, YO, MS, NO, HF, TH, and SN collected the data”. Additionally, the following was added to the Competing Interests: TH is an employee of OncoTherapy Science, Inc. This has been corrected in both the PDF and HTML versions of the Article.

Additionally, the original version of the Supplementary Information associated with this Article contained an error in the ELISPOT method described on page 5, which incorrectly read ‘positive CD8+ T cell responses were identified when the mean number of spot-forming cells per well in response to the respective peptide was ≥ 10 after subtracting background reactivity as determined via a control (non-immunogenic) peptide. The degrees of positivity classified were 10–199 spots = “1+”, 200–299 spots = “2+”, and ≥ 300 spots = “3+”. The correct version states ‘positivity for an antigen-specific CD8+ T-cell response was quantitatively defined according to a modified evaluation tree algorithm based on that described previously^19^. In brief, the number of peptide-specific spots was calculated as the average of triplicates by subtracting the number of spots in the control well from that observed in the well with peptide-pulsed stimulator. Positivity for each antigen-specific CD8+ T-cell response was classified into four grades (−, +, ++, and +++) depending on the number and variability of peptide-specific spots at different responder/stimulator ratios.’ in place of the incorrect text. The original version of this Article omitted a reference to the ELISPOT method in the Supplementary Information. This has been added as reference 19 at the text mentioned above and at the list of references in the Supplementary Information. The HTML has been updated to include a corrected version of the Supplementary Information.

