# Supplementary material for: A VEGF receptor vaccine demonstrates preliminary efficacy in neurofibromatosis type 2
Source: Nat Commun. 2019 Dec 17;10:5758. doi: 10.1038/s41467-019-13640-1 (PMC6917794; doi:10.1038/s41467-019-13640-1)
Supplement: Supplementary file 1 — Supplementary Information [file 41467_2019_13640_MOESM1_ESM.docx]

**Supplementary Information**

**Tamura R, et al.**

The following document contains all of the supplementary information and data for the paper: *A* VEGF receptor vaccine demonstrates preliminary efficacy in Neurofibromatosis type 2

A brief table of contents is below.

**Table of Contents**

**1) Supplementary Text- Supplementary Methods 2**

**2) Table 1- Inclusion and exclusion criteria for** **UMIN000023565　10**

**3) Table 2- Summary of reported cases treated with bevacizumab　11**

**4) Table 3-** **Safety of reported clinical trials using VEGFR1 or VEGFR2 peptide vaccine　12**

**5) Figure 1-Radiographic analysis of other schwannomas and meningiomas after vaccination　13**

**6) Figure 2- Histological analyses of Foxp3 and programmed death-ligand 1 in schwannomas in patients with and without neurofibromatosis type 2 　14**

**7) Figure 3-Histological changes in neurofibromatosis type 2 schwannomas after vaccination16**

**8) Figure 4- Tumor volume segmentation method　17**

**9) Supplementary References　19**

**Supplementary Methods**

**Peptides:** All peptides were synthesized using a standard solid-phase synthesis method and purified via reversed-phase high-performance liquid chromatography (HPLC). The purity (> 95%) and identity of the peptides were determined via analytical HPLC and mass spectrometry, respectively. Vascular endothelial growth factor receptor (VEGFR)1-A24-1084 and VEGFR2-A24-169 peptides (2 mg of each) were emulsified together with 1 ml incomplete Freund’s adjuvant (Montanide ISA-51 VG, SEPPIC, Paris), as were VEGFR1-A02-770 and VEGFR2-A02-773-2L peptides.

**Outcomes and assessments:** Computed tomography (CT) and magnetic resonance imaging (MRI) were used to evaluate clinical responses. Volumetric analysis based on MRI was used to determine the complex shapes of neurofibromatosis type 2 (NF2)-related tumors and quantify their slow growth,^1-3^ because volumetric MRI analysis is known to reflect the actual size of a lesion more closely than linear measurements.^4-9^ In schwannomas with heterogenous intensity or with a cystic component derived from V and VII/VIII nerves located adjacent to each other, a simple system involving manually outlining the target area was better than a semi-automated segmentation method using the SYNAPSE VINCENT imaging system (Fujifilm Medical Co., Tokyo, Japan; see Supplementary Figure 4). In manual segmentation a graphic tool that is a component of AW Workstation and AW Server platforms (AW Server 2, release 5.5, GE Healthcare, Waukesha, WI, USA) was used. After the boundaries of a region of interest are drawn around the lesion on each slice where a lesion is observed, the area is multiplied by the slice thickness (1 mm) to calculate the volume of the lesion within that slice. The volumes of all slices are added to calculate the total lesion volume. It has previously been reported that this method is highly accurate.^7-9^ In the present study all volumetric analysis was performed via 3.0 Tesla MRI, and high-resolution post-contrast T1-weighted MRI sequences were used with a thin slice thickness of 1 mm to measure relatively small tumor volumes. T2-weighted imaging and fluid-attenuated inversion recovery (FLAIR) were not used for the volumetric analysis because edematous change is known to be observed after discontinuation of anti-angiogenic therapy.^10^ Fat suppression was not used because not all patients underwent surgery just prior to vaccination. Fat suppression is required in post-surgical vestibular schwannoma cases to differentiate tumor from fat packing.^3^ Furthermore, tumors with at least 3 imaging slices and well-defined contours were regarded as target regions. To improve accuracy, all analyses were performed via consensus by three authors who were blind to the corresponding clinical information.

Percent change in tumor volume 1 year after vaccination was measured by comparing total tumor volume just prior to the first vaccination with total tumor volume 1 year after the first vaccination.^14^ Similarly, tumor volume 1 year prior to the first vaccination was compared to tumor volume just prior to the first vaccination, and percent change was calculated. Tumor blood volume (TBV) was measured via CT perfusion as a ratio of the relative values to the lesion of interest in normal-appearing white matter.^11-13^ Hearing responses were evaluated via maximum word recognition scores (WRSs).^2,14^ WRSs were assessed using AA-H1 (Rion Co. Ltd.) with 67-S monosyllable wordlists. Patients with WRS ≥ 90 were excluded from hearing evaluation.^2,14^ Thresholds were determined at frequencies of 250, 500, 1000, 2000, 3000, 4000, and 8000 Hz, and pure-tone audiogram values were calculated as the average of thresholds at 500, 1000, 2000, and 3000 Hz. Both ears were examined individually in all patients. Hearing levels in NF2 patients were classified using the American Academy of Otolaryngology - Head and Neck Surgery classification system (class A–D)^15^ and the Tokyo classification system (class A–F).^16^ Peptide-specific immunological responses were assessed using the enzyme-linked immunospot (ELISPOT) assay, which was performed by the contracted research organizations OncoTherapy Science, Inc. (Kanagawa, Japan) and Cancer Precision Medicine (Kanagawa, Japan).^17,18^

**Isolation and stock of peripheral blood mononuclear cells:** Peripheral blood cells were obtained from patients within 2 weeks before the first vaccination, after five vaccinations (at the 3-month timepoint), after eight vaccinations (at the 6-month timepoint), and 12 months after the first vaccination. Peripheral blood mononuclear cells (PBMCs) were isolated immediately using the Ficoll-Paque Plus density gradient solution (GE Healthcare, Little Chalfont, UK), suspended in Cell Banker (Zenoac, Fukushima, Japan), and frozen then stored at -80°C.

**ELISPOT assay and CD8+ T cell responses to peptide stimulation:** In ELISPOT assays, positivity for an antigen-specific CD8+ T-cell response was quantitatively defined according to a modified evaluation tree algorithm based on that described previously.^19^ In brief, the number of peptide-specific spots was calculated as the average of triplicates by subtracting the number of spots in the control well from that observed in the well with peptide-pulsed stimulator. Positivity for each antigen-specific CD8+ T-cell response was classified into four grades (−, +, ++, and +++) depending on the number and variability of peptide-specific spots at different responder/stimulator ratios. The induction of cytotoxic T lymphocytes (CTLs) by the vaccine was deemed to have occurred when positivity increased after vaccination.

**Immunohistochemical analysis:** Histopathological analyses were performed on 4-µm sections of formalin-fixed paraffin-embedded tissue samples from 25 tumors derived from 11 NF2 patients and 21 tumors derived from 21 non-NF2 patients. Four of the 25 tumor samples were paired pre- and post-vaccination samples obtained from two NF2 patients. A trigeminal schwannoma was partially removed at the time of an intracranial hemorrhage 5 months after the last vaccination in case 1. In case 2, although a skin schwannoma had stabilized it was completely removed 11 months after the last vaccination at the patient’s request for solely cosmetic reasons. The expression of vascular endothelial growth factor (VEGF)-A, VEGFR-1 (flt-1), and VEGFR-2 (KDR/flk-1) was examined via immunohistochemistry using an anti-VEGF-A mouse monoclonal antibody (1:200, JH121, 05-443, Merick Millipore, Darmstadt, Germany), anti-VEGFR-1 goat polyclonal antibody (1:100, Flt-1, AF321, R&D Systems, MN, USA), and anti-VEGFR-2 rabbit polyclonal antibody (1:600, 55B11, 2479, Cell Signaling Technology, Tokyo, Japan). CD34 and platelet-derived growth factor receptor-beta (PDGFR-β) expression were examined via immunohistochemistry using an anti-CD34 mouse monoclonal antibody (1:100, NU-4A1, 413361, Nichirei Biosciences Inc., Tokyo, Japan) and an anti-PDGFR-β rabbit monoclonal antibody (1:50, Y92, ab32570, Abcam, MA, USA).^1^ Sections were also examined for key molecules and cells in the tumor immune-microenvironment, including programmed cell death ligand 1 (PD-L1; an immune checkpoint molecular marker), CD8 (a CTL marker), and the regulatory T cell (Treg) marker Foxp3. Immunohistochemistry was performed with anti-PD-L1 rabbit monoclonal antibody (1:500, 28-8, ab205921, Abcam), anti-CD8 mouse monoclonal antibody (1:100, 144B, ab17147, Abcam), and anti-Foxp3 rabbit polyclonal antibody (1:100, 236A/E7, ab54501, Abcam). For the assessment of apoptosis, paired pre- and post-vaccination sections were examined via immunohistochemistry using anti-cleaved caspase 3 rabbit monoclonal antibody (1:200, ASP 175, 9664, Cell Signaling Technology). Antigen retrieval was performed in citrate buffer (pH 6) for VEGFR1 and Foxp3, and in Tris buffer (pH 9) for VEGF-A, VEGFR2, CD34, PDGFR-β, PD-L1, CD8, and cleaved caspase 3. Microwave irradiation was used for all antigens except PD-L1, for which autoclaving was used. The products were visualized via a peroxidase-diaminobenzidine reaction. For the assessment of microvessel density (MVD), tissue sections were screened after CD34 immunohistochemistry staining under low-power magnification (x40), and the five most vascularized areas (hot-spots) were selected. Microvessel counting was performed automatically in these areas under high-power magnification microscopy (x200, 0.95 mm^2^) (Biorevo BZ-9000, KEYENCE). The diameter of vessels was also measured automatically in these same areas.^20^ For the assessment of CD8 and Foxp3, the stained sections were screened under low-power magnification microscopy (x40) and five hot-spots were selected. We then counted the numbers of positive cells in these areas under high-power magnification microscopy (x400, 0.47 mm^2^). PD-L1 expression was scored as a percentage of tumor cells expressing PD-L1 (≥ 50% = 3+, ≥ 5% but < 50% = 2+, ≥ 1% but < 5% = 1+, and < 1% = 0).^21^ All expression levels were assessed via consensus by four authors who were blinded to the corresponding clinical information (RT, KO, YM, and MT).

**Immunofluorescence analysis:** Immunofluorescence triple staining for VEGFR1, VEGFR2, and PDGFR-β expression and VEGFR1, VEGFR2, and CD8 expression was performed to evaluate vascular characteristics. Tissue sections were incubated with antibodies against VEGFR1 (1:100, AF321, goat polyclonal IgG, R&D Systems), VEGFR2 (1:100, EIC, mouse monoclonal IgG, Abcam), and PDGFR-β (1:50, Y92, rabbit monoclonal IgG, Abcam), or VEGFR1 (1:100, AF321, goat polyclonal IgG, R&D Systems), VEGFR2 (1:600, 55B11, rabbit monoclonal IgG, Cell Signaling Technology), and CD8 (1:200, 144B, mouse monoclonal IgG, Abcam) overnight at 4˚C. Appropriate secondary antibodies, Alexa Fluor 488 donkey anti-mouse IgG, Alexa Fluor 568 donkey anti-goat IgG, or Alexa Fluor 647 donkey anti-rabbit IgG were used (1:200). Last, the sections were mounted with mounting medium for fluorescence with 4’,6-diamidino-2-phenylindole (DAPI; Santa Cruz Biotechnology, TX, USA). Cell images were captured via fluorescence microscopy (Biorevo BZ-9000, KEYENCE, Osaka, Japan).

**RNA extraction, cDNA synthesis, and quantitative real-time PCR:** For quantitative real-time PCR (qPCR), RNA was isolated from 10-μm sections of formalin-fixed, paraffin-embedded tissue in 46 samples using the NucleoSpin Total　RNA FFPE XS Kit (Macherey-Nagel, , Düren, Germany). cDNA was subsequently obtained using the SuperScript II First Strand Synthesis System with random hexamers (Invitrogen). qPCR was performed with 10 μl Applied Biosystems SYBR Green reagent (Thermo Fisher Scientific, Massachusetts, USA), 2 μl synthesized cDNA , 2 μl of the same primers that were used in the standard PCR, and 6 μl of molecular grade water in a total volume of 20 μl. qPCR was performed in triplicate using an ABI StepOnePlus real-time PCR machine (Applied Biosystems, Foster, USA). Amplification was performed under the following conditions: 10 min at 95˚C, 55 cycles of 95˚C for 15 s, 60˚C for 60 s. All experiments included negative controls (non-template water instead of cDNA). qPCR data were analyzed using the comparative CT method. Briefly, the difference in cycle threshold, ΔCT, was determined as the difference between the tested gene and human glyceraldehyde 3-phosphate dehydrogenase (GAPDH). Data were then normalized to GAPDH cDNA. The fold change was calculated as 2^-ΔΔCT^.^22^ The following primer sequences were utilized:

VEGF-A forward (fwd): GAGATGAGCTTCCTACAGCAC

VEGF-A reverse (rev): TCACCGCCTCGGCTTGTCACAT

VEGFR1 fwd: CAGGCCCAGTTTCTGCCATT

VEGFR1 rev: TTCCAGCTCAGCGTGGTCGTA

VEGFR2 fwd: CCAGCAAAAGCAGGGAGTCTGT

VEGFR2 rev: TGTCTGTGTCATCGGAGTGATATCC

FOXP3 fwd: GGCCCTTCTCCAGGACAGA

FOXP3 rev: GCTGATCATGGCTGGGTTGT

GAPDH fwd: TGAACGGGAAGCTCACTGG

GAPDH rev: TCCACCACCCTGTTGCTGTA

**Supplementary Table 1. Inclusion and exclusion criteria for UMIN000023565.**

| **Inclusion criteria** | **Exclusion criteria** |
| --- | --- |
| - NF2 patients diagnosed with progressive schwannoma - Announcement of a diagnosis - Positive genomic DNA typing for HLA-A*2402, 0201, 0206 and 0207 (HLA Laboratory, Kyoto, Japan) - Age between 12 and 79 years - No surgery, irradiation, or chemotherapy in the 4 weeks prior to enrolment in the study - Life expectancy >3 months - Written informed consents obtained   **Laboratory test values prior to vaccination**   - Neutrophil count ≥1000/mm^3^ - Platelet count ≥50,000/mm^3^ - Hemoglobin level ≥8.0g/dl, a - Aspartate aminotransferase and alanine aminotransferase ≤ 4.0x the institutional normal upper limits - Total bilirubin ≤ 1.5x the institutional normal upper limits - Creatinine ≤ 2.0mg/dl - No uncontrollable pleural, peritoneal, or cardiac effusion | - The presence of uncontrollable severe infectious diseases - Adverse event of National Cancer Institute - Common Toxicity Criteria grade 3 or 4 - Unable to take anything orally over 24 hours - Other uncontrolled malignant disease - Myeloproliferative disease - Prior allogeneic hematopoietic stem cell transplantation - Active autoimmune disease - Severe drug allergy - Concurrent treatment with steroids or immunosuppressive agents - Pregnancy or planning to become pregnant during the study period - Psychiatric disorder - Unhealed wound - Decision of unsuitability by the principal investigator or the physician in charge. |

**Supplementary Table 2. Summary of reported cases treated with bevacizumab**

|  | **Cases** | **Dose**  **(mg/kg)** | **Median duration**  **(month)** | **RR (%)*** | **Patient with RR**  **(%)** | **Patient with HR**  **(%)** | **Not effective case** | **Toxicity** |
| --- | --- | --- | --- | --- | --- | --- | --- | --- |
| **Plotkin et al.**  **2009** | 10 | 5 | 12 | >20 | 60 | 57 | 1 | N/A |
| **Mautner et al.**  **2010** | 2 | 5 | 12 | N/A | 100 | 50 | - | HT |
| **Subbiah et al.**  **2012** | 2 | 5 | 9.5 | N/A | 0 | 0 | - | N/A |
| **Plotkin et al.**  **2012** | 31 | 5 | 11.5 | >20 | 55 | 57 | 6 | N/A |
| **Eminowicz et al. 2012** | 1 | 5 | 3.5 | N/A | 100 | 0 | - | N/A |
| **Blakeley et al.**  **2014** | 1 | 10^#^ | Continuously | N/A | 100 | 0 | - | N/A |
| **Versleijen et al.**  **2014** | 1 | 5 | N/A | N/A | 100 | 100 | - | N/A |
| **Alanin et al.**  **2015** | 12 | 10 | 22 | >20 | 50 | 50 | - | ICH |
| **Liu et al.**  **2016** | 1 | 3.3^†^ | 3 | N/A | 100 | 100 | - | N/A |

HR, hearing response; HT, hypertension; ICH, intracerebral hemorrhage; N/A, not available; RR, radiographic response

*Volumetric percent change defined as a radiographic response

^#^10 mg/kg intravenously every 2 weeks for 6 months, followed by 7.5 mg/kg intravenously every 3 weeks continuously

^†^3.3 mg/kg every 2 weeks for a period of 3 months

**Supplementary Table 3. Safety of reported clinical trials using VEGFR1 or VEGFR2 peptide vaccines**

| **Author, year** | **Phase** | **Disease** | **Number** | **HLA** | **Peptide** | **Chemo** | **Toxicity** |
| --- | --- | --- | --- | --- | --- | --- | --- |
| **Miyazawa,**  **et al. 2010** | I | pancreatic cancer | 18 | 24:02 | VEGFR2 (OTS-102) | gemcitabine | Grade 4:0 Grade 3:1 (duodenal hemorrhage), 2 (AST and ALT↑), 2 (leukopenia) |
| **Masuzawa,**  **et al. 2012** | I/II | gastric cancer | 22 | 24:02 | VEGFR1 and 2  (OCV-101 ‧ OTS-102) | S-1 + cisplatin | Grade 3 or 4: leukopenia and anemia |
| **Yoshimura,**  **et al. 2013** | I | metastatic kidney cancer | 18 | 24:02 02:01 | VEGFR1 (OCV-101 ‧ MPS-400) |  | Grade 3 or 4:0 |
| **Hazama,**  **et al. 2014** | I | colorectal cancer | 19 | 24:02 | VEGFR1 and 2, (OCV-101 ‧ OTS-102), RNF43, TOMM34, KOCI | ― | Grade 3 or 4:0 |
| **Hazama,**  **et al. 2014** | II | colorectal cancer | 96 | 24:02 | VEGFR1 and 2 (OCV-101 ‧ OTS-102), RNF43, TOMM34, KOCI | mFOLFOX6,XELOX | Grade 4:5 (interstitial pneumonia, neutropenia) 1: embolism |
| **Iinuma,**  **et al. 2014** | I | esophageal cancer | 11 | 24:02 | VEGFR1 and 2 (OCV-101 ‧ OTS-102), TTK protein kinase (TTK), up-regulated lung cancer 10 (URLC10), insulin-like growth factor-II mRNA binding protein 3 (KOC1) | 5-FU + cisplatin | Grade 4:0 Grade 3: leukopenia, anemia, |
| **Shibao,**  **et al. 2018** | I/II | glioblastoma | 8 | 24:02 | VEGFR1 and 2 (OCV-101 ‧ OTS-102) | ― | Grade 3:1 (injection site ulcer) |
| **Kikuchi,**  **et al. 2019** | I/II | glioblastoma | 10 | 24:02 | VEGFR1 and 2 (OCV-101 ‧ OTS-102), multiple glioma oncoantigen (LY6K, DEPDC1, KIF20A, and FOXM1) | ― | Grade 3 or 4:0 |

Chemo, Chemotherapy; CTL, cytotoxic T lymphocyte; VEGFR, vascular endothelial growth factor receptor; 5-FU, 5-Fluorouracil


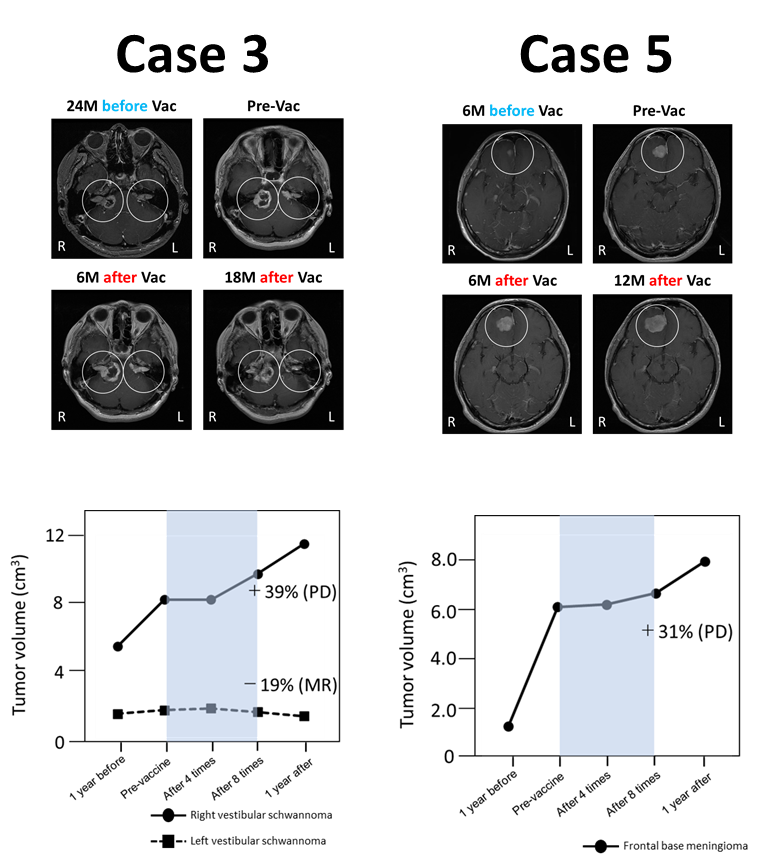


**Supplementary Figure 1. Radiographic analysis of schwannomas (cases 3) and meningioma (case 5) after vaccination.**

White circles indicate tumors targeted for volumetric analysis. In case 3 MR was observed in the left vestibular schwannoma, but PD was evident in the right cystic vestibular schwannoma. The growth of the cystic schwannoma in case 3 had ceased 12 months after the first vaccination. In case 5 PD was evident in the frontal base meningioma, which grew rapidly prior to vaccination.

L, left; M, months; MR, minor response; PD, progressive disease; R, right; Vac, vaccine; Pre-Vac, the day of the first vaccination


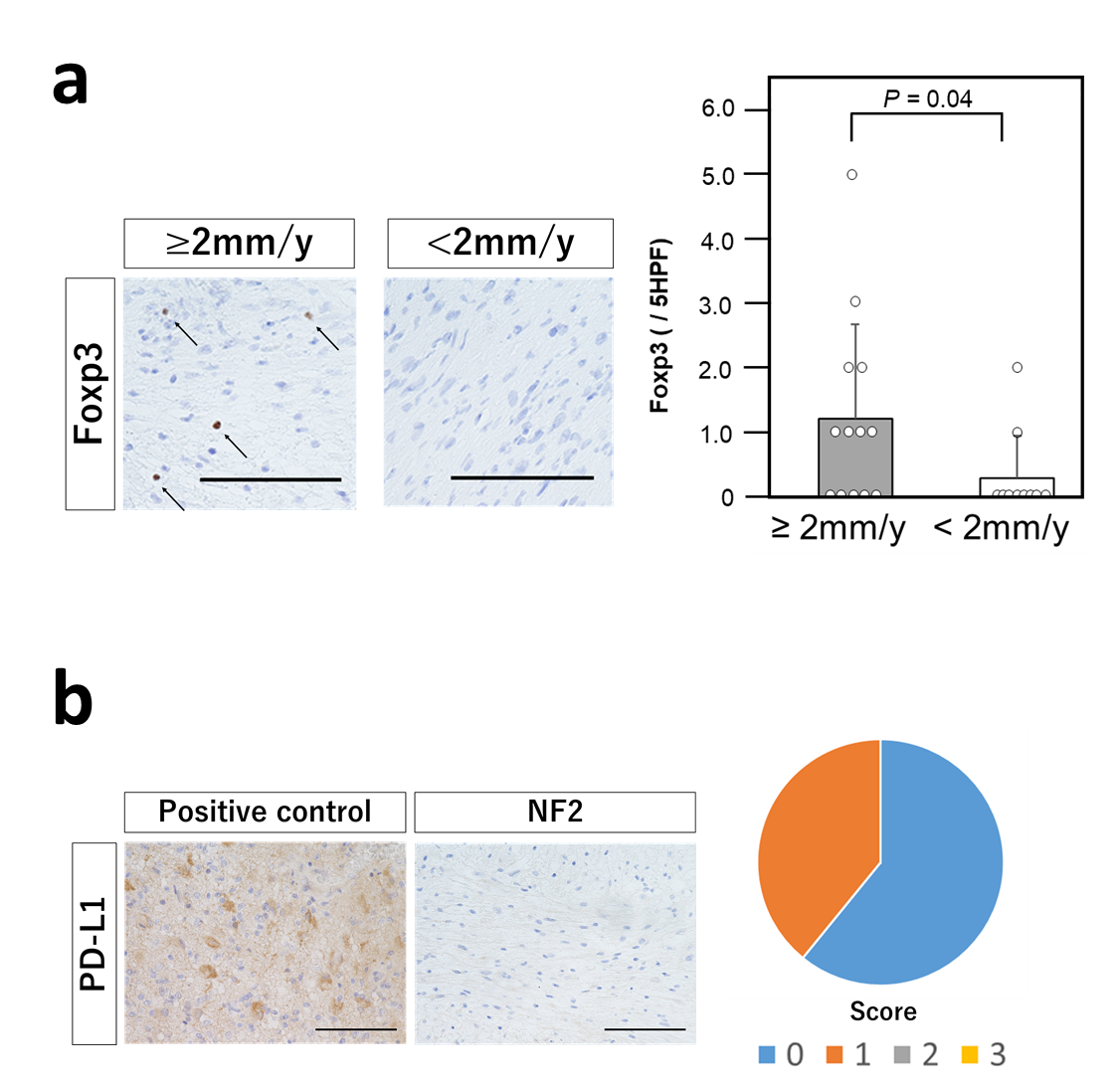


**Supplementary Figure 2. Histological analyses of Foxp3 and programmed cell death ligand 1 (PD-L1) expression in schwannomas in neurofibromatosis type 2 (NF2) and non-NF2 patients.**

(a) Foxp3 expression in NF2 schwannomas with different growth patterns (≥ 2 mm/year and < 2 mm/year). The numbers of Foxp3 positive cells per 5 high-power magnification microscopy fields was significantly higher in the growth ≥ 2 mm/year group than it was in the growth < 2 mm/year group. *P* values were determined by student’s *t*-test. Black arrows indicate Foxp3-positive cells.

Original magnification ×400, magnification bar = 100 μm. The mean (bar) ± SD. (error bars) is shown (≥2mm/year, n=13; < 2 mm/year, n=10).

(b) PD-L1 expression in NF2 schwannomas. PD-L1 expression was scored as a percentage of tumor cells expressing PD-L1 (≥ 50% = 3+, ≥ 5% but < 50% = 2+, ≥ 1% but < 5% = 1+, and < 1% = 0). Of the total of 23 tumors, 14 yielded PD-L1 scores of 0, and 9 yielded scores of 1.

Original magnification ×400, magnification bar = 100 μm.

5HPF, 5 high-power magnification microscopy fields; NF2, neurofibromatosis type 2;

PD L1, programmed cell death ligand 1; y, year

**
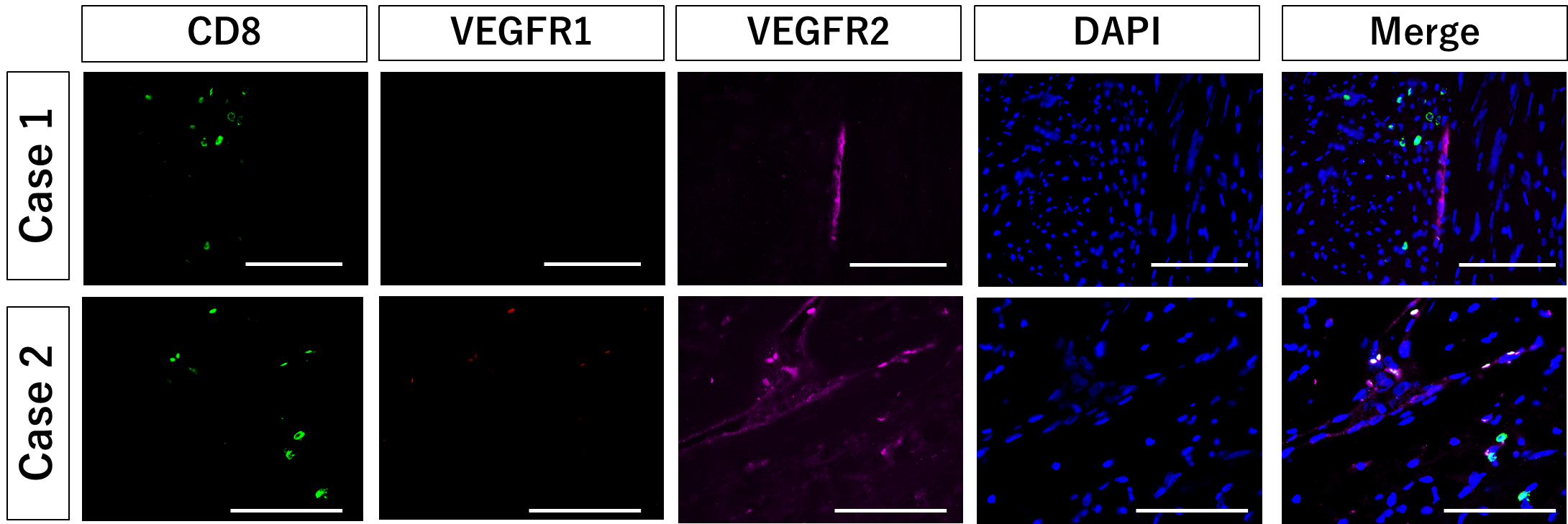
**

**Supplementary Figure 3. Histological changes in neurofibromatosis type 2 schwannomas after vaccination.**

Immunofluorescence analysis after vaccination. Some vessels with strong vascular endothelial growth factor receptor (VEGFR) expression are evident, and CD8-positive cells are present around the vessels with weak VEGFR expression after vaccination. Original magnification ×400, magnification bar = 100 μm.

DAPI, 4′,6-diamidino-2-phenylindole; VEGFR, vascular endothelial growth factor receptor


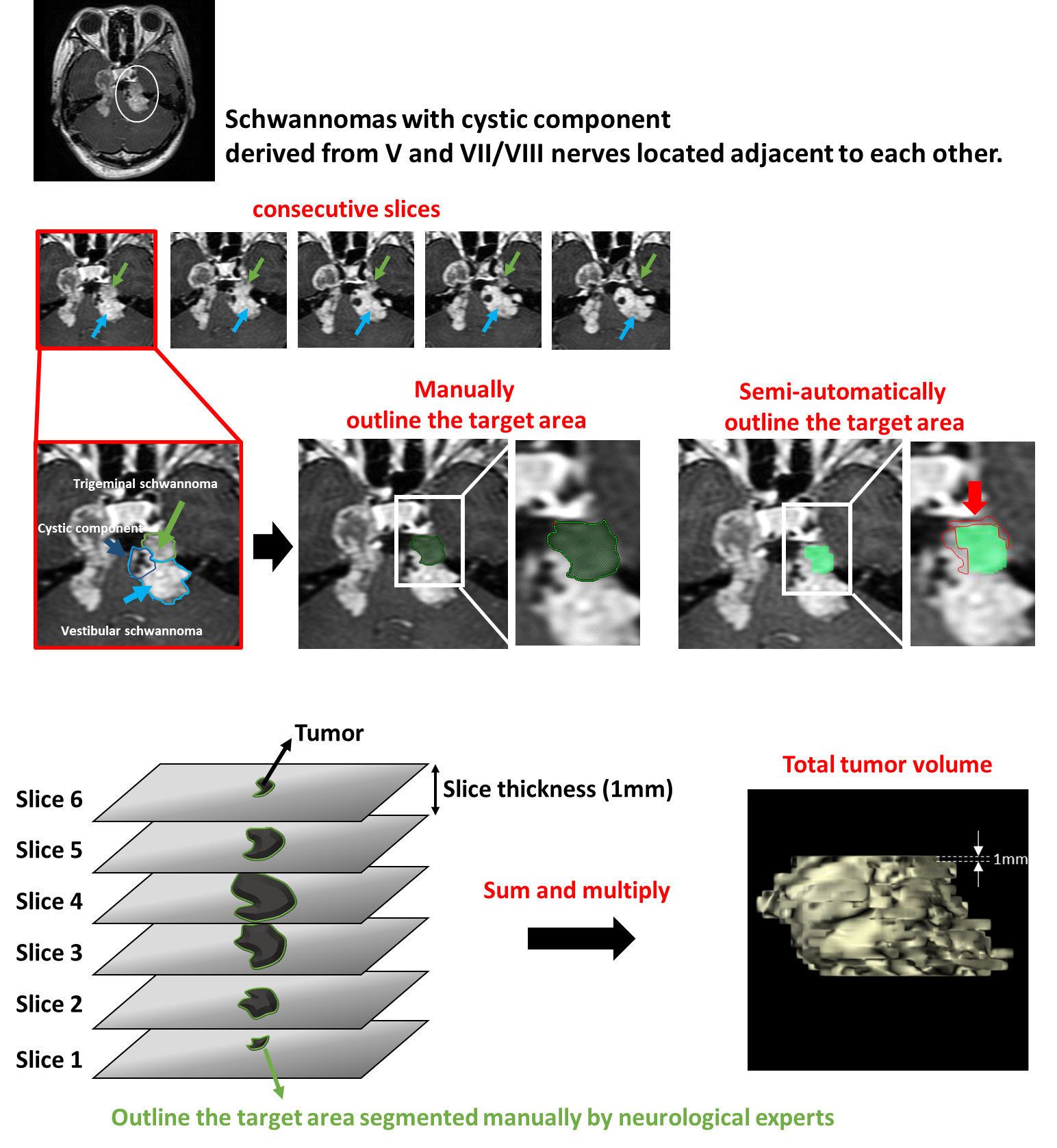


**Supplementary Figure 4. Tumor volume segmentation method.**

To evaluate tumor volume, comparative analysis of the semi-automatic segmentation tool function of the SYNAPSE VINCENT imaging system (Fujifilm Medical Co., Tokyo, Japan) and the simple system using a graphic tool component of AW Workstation and AW Server platforms (AW Server 2, release 5.5, GE Healthcare, Waukesha, WI, USA) to manually outline the target area on gadolinium-enhanced T1-weighted images (see Supplementary Methods) was performed. In manual segmentation a region of interest was drawn around the lesion boundaries on each slice where the lesion was observed. The area was multiplied by the slice thickness (1 mm) to calculate the volume of the lesion within that slice, and the volumes from all slices were added to calculate the total lesion volume. In cases of schwannomas with heterogenous intensity or with a cystic component derived from V and VII/VIII nerves located adjacent to each other the simple system involving manually outlining the target area was better than the semi-automated segmentation method. The panels outlined in red depict inaccurate determination of the area of trigeminal schwannomas when the semi-automated segmentation method was used.

**Supplementary References**

1.Tamura R, et al. Histopathological vascular investigation of the peritumoral brain zone of glioblastomas. *J Neurooncol* **136**:233- 241 (2018)

2. Blakeley JO, et al. Efficacy and Biomarker Study of Bevacizumab for Hearing Loss Resulting From Neurofibromatosis Type 2-Associated Vestibular Schwannomas. *J Clin Oncol* **34**:1669-1675 (2016)

3.Dombi E, et al. REiNS International Collaboration. Recommendations for imaging tumor response in neurofibromatosis clinical trials. *Neurology* **81**:S33- 40 (2013).

4.Solomon J, et al. Automated detection and volume measurement of plexiform neurofibromas in neurofibromatosis 1 using magnetic resonance imaging. *Comput Med Imaging Graph* 28:257-265 (2004)

5.Harris GJ, et al. Three-dimensional volumetrics for tracking vestibular schwannoma growth in neurofibromatosis type II. *Neurosurgery* **62**:1314-1319 (2008)

6.Weizman L, et al. Interactive segmentation of plexiform neurofibroma tissue: method and preliminary performance evaluation. *Med Biol Eng Comput.* **50**:877-884 (2012)

7. Goldmacher GV, et al. The use of tumour volumetrics to assess response to therapy in anticancer clinical trials. *Br J Clin Pharmacol* **73**:846-854 (2012)

8. Pupulim LF, et al. Volumetric measurement of hepatic tumors: Accuracy of manual contouring using CT with volumetric pathology as the reference method. *Diagn Interv Imaging* **99**:83-89 (2018)

9. Veeraraghavan H, et al. Appearance Constrained Semi-Automatic Segmentation from DCE-MRI is Reproducible and Feasible for Breast Cancer Radiomics: A Feasibility Study. *Sci Rep* **8**:4838 (2018)

10. Moserle L, et al. Antiangiogenic therapies: going beyond their limits. *Cancer Discov* **4**:31-41 (2014)

11.Welker K, et al. ASFNR recommendations for clinical performance of MR dynamic susceptibility contrast perfusion imaging of the brain. *AJNR Am J Neuroradiol* **36**:E41- 51 (2015)

12.Xyda A, et al. Brain volume perfusion CT performed with 128-detector row CT system in patients with cerebral gliomas: a feasibility study. *Eur Radiol* **21**:1811- 1819 (2011)

13.Beppu T, et al. Prediction of malignancy grading using computed tomography perfusion imaging in nonenhancing supratentorial gliomas. *J Neurooncol* **103**:619- 627 (2011)

14.Plotkin SR, et al. Hearing and facial function outcomes for neurofibromatosis 2 clinical trials. *Neurology* **81**:S25- 32 (2013)

15.American Academy of Otolaryngology-Head and Neck Surgery, author. Committee on hearing and equilibrium guidelines for the evaluation of hearing preservation in acoustic neuroma (vestibular schwannoma). *Otolaryngol Head Neck Surg* **113**:179- 180 (1995)

16. Kanzaki J, et al. New and modified reporting systems from the Consensus Meeting on Systems for Reporting Results in Vestibular Schwannoma. *Otol Neurotol* **24**:642- 649 (2003)

17.Ranieri E, et al. CTL ELISPOT assay. *Methods Mol Biol* **1186**:75- 86 (2014)

18.Shibao S, et al. A pilot study of peptide vaccines for VEGF receptor 1 and 2 in patients with recurrent/progressive high grade glioma. *Oncotarget* **9**:21569- 21579 (2018)

19. Kono K, et al. Multicenter, phase II clinical trial of cancer vaccination for advanced esophageal cancer with three peptides derived from novel cancer-testis antigens. J Transl Med 10:141 (2012)

20.Tamura R, et al. Histopathological investigation of glioblastomas resected under bevacizumab treatment. *Oncotarget* **7**:52423- 52435 (2016)

21.Fehrenbacher L, et al. Atezolizumab versus docetaxel for patients with previously treated non-small-cell lung cancer (POPLAR): a multicenter, open-label, phase 2 randomized controlled trial. *Lancet* **387**:1837- 1846 (2016)

22.Boeckx C, et al. Expression analysis on archival material: comparison of 5 commercially available RNA isolation kits for FFPE material. *Diagn Mol Pathol* **20**:203- 211 (2011)
