# Supplementary material for: A VEGF receptor vaccine demonstrates preliminary efficacy in neurofibromatosis type 2
Source: Nat Commun. 2019 Dec 17;10:5758. doi: 10.1038/s41467-019-13640-1 (PMC6917794; doi:10.1038/s41467-019-13640-1)
Supplement: Supplementary file 3 — Reporting Summary [file 41467_2019_13640_MOESM3_ESM.pdf]

nature research

Corresponding author(s): Masahiro Toda

Last updated by author(s): Oct 24, 2019

Reporting Summary

Nature Research wishes to improve the reproducibility of the work that we publish. This form provides structure for consistency and transparency in reporting. For further information on Nature Research policies, see Authors & Referees and the Editorial Policy Checklist.

Statistics

For all statistical analyses, confirm that the following items are present in the figure legend, table legend, main text, or Methods section.

n/a	Confirmed	
☐	☒	The exact sample size (*n*) for each experimental group/condition, given as a discrete number and unit of measurement
☐	☒	A statement on whether measurements were taken from distinct samples or whether the same sample was measured repeatedly
☒	☐	The statistical test(s) used AND whether they are one- or two-sided<br>*Only common tests should be described solely by name; describe more complex techniques in the Methods section.*
☐	☒	A description of all covariates tested
☐	☒	A description of any assumptions or corrections, such as tests of normality and adjustment for multiple comparisons
☐	☒	A full description of the statistical parameters including central tendency (e.g. means) or other basic estimates (e.g. regression coefficient) AND variation (e.g. standard deviation) or associated estimates of uncertainty (e.g. confidence intervals)
☒	☐	For null hypothesis testing, the test statistic (e.g. *F*, *t*, *r*) with confidence intervals, effect sizes, degrees of freedom and *P* value noted<br>*Give P values as exact values whenever suitable.*
☒	☐	For Bayesian analysis, information on the choice of priors and Markov chain Monte Carlo settings
☒	☐	For hierarchical and complex designs, identification of the appropriate level for tests and full reporting of outcomes
☐	☒	Estimates of effect sizes (e.g. Cohen's *d*, Pearson's *r*), indicating how they were calculated

Our web collection on statistics for biologists contains articles on many of the points above.

Software and code

Policy information about availability of computer code

Data collection	All-in-one Fluorescence Microscope software (KEYENCE, BZ-H3A) was used for immunohistochemistry image aquisition.<br>For qPCR analyses, we used StepOne Software v2.3 (Applied Biosystems StepOne™ and StepOnePlus™ Real-Time PCR Systems).<br>For the analysis of tumor volume, AW Workstation and AW Server platforms (AW server 2, release 5.5, GE Healthcare, Waukesha, Wis) and SYNAPSE VINCENT imaging system (Fujifilm Medical Co., Tokyo, Japan) were used on gadolinium-enhanced MRI T1WI images.
Data analysis	To evaluate immunohistochemical findings (microvessel density, vessel diameter and positive cell count), we used BZ-9000 Fluorescence Microscope (KEYENCE), and all-in-one Fluorescence Microscope software (KEYENCE, Analysis application hybrid cell count; BZ-H3C).<br>Statistics was completed using IBM SPSS Statistics.

For manuscripts utilizing custom algorithms or software that are central to the research but not yet described in published literature, software must be made available to editors/reviewers. We strongly encourage code deposition in a community repository (e.g. GitHub). See the Nature Research guidelines for submitting code & software for further information.

Data

Policy information about availability of data

All manuscripts must include a data availability statement. This statement should provide the following information, where applicable:

- Accession codes, unique identifiers, or web links for publicly available datasets
- A list of figures that have associated raw data
- A description of any restrictions on data availability

All data supporting the findings of this study are available within the article and its Supplementary Information Files and from the corresponding author on reasonable request.

Field-specific reporting

Please select the one below that is the best fit for your research. If you are not sure, read the appropriate sections before making your selection.

☒ Life sciences ☐ Behavioural & social sciences ☐ Ecological, evolutionary & environmental sciences

For a reference copy of the document with all sections, see nature.com/documents/nr-reporting-summary-flat.pdf

Life sciences study design

All studies must disclose on these points even when the disclosure is negative.

Sample size

7 patients in the clinical trial and 49 human schwannoma specimens.
Interim analysis is conducted to evaluate whether to stop or modify the study for futility and/or safety issues, which is performed by aforementioned Independent Data Monitoring Committee.
In this study, grade 5 intracranial hemorrhaging was observed in one patient 5 months after the last vaccination. Detailed image analysis revealed dural arteriovenous fistula (dAVF) around the left temporal lobe at the time of hemorrhage, and also prior to vaccination. dAVF may have been the origin of hemorrhage. The aforementioned Independent Data Monitoring Committee concluded that the experimental vaccine was not likely to be related to this adverse event. Although the number of cases was small, the safety of the vaccine was evident in these 7 patients with progressive NF2 with schwannomas. Therefore, intermediate reporting was submitted to Nature communications.

Data exclusions

Exclusion criteria of the clinical trial
- The presence of uncontrollable severe infectious diseases
- Adverse event of National Cancer Institute - Common Toxicity Criteria grade 3 or 4
- Unable to take anything orally over 24 hours
- Other uncontrolled malignant disease
- Myeloproliferative disease
- Prior allogeneic hematopoietic stem cell transplantation
- Active autoimmune disease
- Severe drug allergy
- Concurrent treatment with steroids or immunosuppressive agents
- Pregnancy or planning to become pregnant during the study period
- Psychiatric disorder
- Unhealed wound
- Decision of unsuitability by the principal investigator or the physician in charge.

These exclusion criteria were established based on our previous clinical trials using VEGFR peptides for the patients with malignant glioma. These previous clinical trials have been previously published [Shibao S. Oncotarget. 2018; Kikuchi R. J Clin Med. 2019].

Replication

All experimental findings were reproducible (at least duplicate).

Randomization

This study was a non-randomized, open label, phase I and II clinical trial of VEGFRs peptide vaccination for progressive NF2. The prevalence of NF2 is reportedly approximately 1 in 25,000 to 1 in 33,000. In addition, HLA type matching also restricted the number of patients enrolled. NF2 patients showed various clinical courses. Therefore, all clinical courses of post-vaccination were compared with that of pre-vaccination in each patient.

Blinding

All analyses were assessed by consensus of four authors with blinded clinical information (RT, YM, KK, and KO).

Reporting for specific materials, systems and methods

We require information from authors about some types of materials, experimental systems and methods used in many studies. Here, indicate whether each material, system or method listed is relevant to your study. If you are not sure if a list item applies to your research, read the appropriate section before selecting a response.

Materials & experimental systems

n/a	Involved in the study	
☐	☒	Antibodies
☒	☐	Eukaryotic cell lines
☒	☐	Palaeontology
☒	☐	Animals and other organisms
☐	☒	Human research participants
☐	☒	Clinical data

Methods

n/a	Involved in the study	
☒	☐	ChIP-seq
☒	☐	Flow cytometry
☐	☒	MRI-based neuroimaging

Antibodies

Antibodies used

anti-VEGF-A mouse monoclonal antibody (1:200, JH121, 05-443, Merick Millipore, Darmstadt, Germany)
anti-VEGFR-1 goat polyclonal antibody (1:100, Flt-1, AF321, R&D Systems, MN, USA)
anti-VEGFR-2 rabbit polyclonal antibody (1:600, 55B11, 2479, Cell Signaling Technology, Tokyo, Japan)
anti-CD34 mouse monoclonal antibody (1:100, NU-4A1, 413361, Nichirei Biosciences Inc., Tokyo, Japan)
anti-PDGFR-β rabbit monoclonal antibody (1:50, Y92, ab32570, Abcam, MA, USA)
anti-PD-L1 rabbit monoclonal antibody (1:500, 28-8, ab205921, Abcam)
anti-VEGFR2 mouse monoclonal antibody (1:100, EIC, ab9530, Abcam)
anti-CD8 mouse monoclonal antibody (1:100, 144B, ab17147, Abcam)
anti-Foxp3 rabbit polyclonal antibody (1:100, 236A/E7, ab54501, Abcam)
anti-cleaved caspase 3 rabbit monoclonal antibody (1:200, ASP 175, 9664, Cell Signaling Technology)

Commercially available antibodies were used and are described appropriately in the method section.

Validation

Antibodies were commercially available and validated by the vendors.
• anti-VEGF-A mouse monoclonal antibody (1:200, JH121, 05-443, Merick Millipore, Darmstadt, Germany)：http://www.merckmillipore.com/JP/en/product/Anti-VEGF-Antibody-clone-JH,MM_NF-05-443
• anti-VEGFR-1 goat polyclonal antibody (1:100, Flt-1, AF321, R&D Systems, MN, USA)：https://www.rndsystems.com/products/human-vegfr1-flt-1-antibody_af321
• anti-VEGFR-2 rabbit polyclonal antibody (1:600, 55B11, 2479, Cell Signaling Technology, Tokyo, Japan)：https://en.cellsignal.jp/products/primary-antibodies/vegf-receptor-2-55b11-rabbit-mab/2479
• anti-CD34 mouse monoclonal antibody (1:100, NU-4A1, 413361, Nichirei Biosciences Inc., Tokyo, Japan)：https://www.nichirei.co.jp/bio/products/immunity/first_koutai.html#413111
• anti-PDGFR-β rabbit monoclonal antibody (1:50, Y92, ab32570, Abcam, MA, USA)：https://www.abcam.co.jp/pdgfr-beta-antibody-y92-c-terminal-ab32570.html
• anti-PD-L1 rabbit monoclonal antibody (1:500, 28-8, ab205921, Abcam)：https://www.abcam.co.jp/pd-l1-antibody-28-8-ab205921.html
• anti-VEGFR2 mouse monoclonal antibody (1:100, EIC, ab9530, Abcam)：https://www.abcam.co.jp/vegf-receptor-2-antibody-eic-ab9530.html
• anti-CD8 mouse monoclonal antibody (1:100, 144B, ab17147, Abcam)：https://www.abcam.co.jp/cd8-alpha-antibody-144b-ab17147.html
• anti-Foxp3 rabbit polyclonal antibody (1:100, 236A/E7, ab54501, Abcam)：https://www.abcam.co.jp/foxp3-antibody-236ae7-ab20034.html
• anti-cleaved caspase 3 rabbit monoclonal antibody (1:200, ASP 175, 9664, Cell Signaling Technology)：https://en.cellsignal.jp/products/primary-antibodies/cleaved-caspase-3-asp175-5a1e-rabbit-mab/9664

Human research participants

Policy information about studies involving human research participants

Population characteristics

• NF2 patients diagnosed with progressive schwannoma
• Both genders (3 males and 4 females in this study)
• Announcement of a diagnosis
• Positive genomic DNA typing for HLA-A*2402, 0201, 0206 and 0207 (HLA Laboratory, Kyoto, Japan)
• Age between 12 and 79 years
• No surgery, irradiation, or chemotherapy in the 4 weeks prior to enrolment in the study
• Life expectancy >3 months
• Written informed consents obtained
• Laboratory test values prior to vaccination
Neutrophil count ≥1000/mm3
Platelet count ≥50,000/mm3
Hemoglobin level ≥8.0g/dl
Aspartate aminotransferase and alanine aminotransferase ≤ 4.0x the institutional normal upper limits
Total bilirubin ≤ 1.5x the institutional normal upper limits
Creatinine ≤ 2.0mg/dl
No uncontrollable pleural, peritoneal or cardiac effusion

Recruitment

The trial was registered at University hospital Medical Information Network (UMIN). UMIN000023565, Open public recruiting

Ethics oversight

All protocols were approved by the Keio University Ethics Committee (number: 20150421), and conducted in accordance with the Helsinki declaration on experimentation on human subjects. Adherence to the trial protocol and accuracy of the completed case report forms and the electronic datasets were assessed at a minimum of three external monitoring visits. The Keio University Clinical and Translational Center, which acts as an independent academic contract research organization, performed the monitoring.

Note that full information on the approval of the study protocol must also be provided in the manuscript.

Clinical data

Policy information about clinical studies

All manuscripts should comply with the ICMJE guidelines for publication of clinical research and a completed CONSORT checklist must be included with all submissions.

Clinical trial registration	The trial was registered at UMIN (UMIN000023565).
Study protocol	Full trial protocol was accessed in UMIN.
Data collection	August 17, 2016 - March 31, 2021
Outcomes	Primary outcome was the safety of the vaccine. Secondary outcomes were clinical efficacy parameters including tumor size, hearing ability, and immunological response. Toxicity was assessed using the Common Terminology Criteria for Adverse Events version 4.0 at each visit. To evaluate clinical responses, computed tomography (CT), magnetic resonance imaging (MRI) and hearing examinations were performed within 2 weeks before the first vaccination, after five vaccinations (at the 3-month timepoint), after eight vaccinations (at the 6-month timepoint), and 12 months after the first vaccination. Individual tumor size was volumetrically measured via gadolinium-enhanced imaging. In the present study, volumetric reductions of > 20% were deemed to indicate a radiological response.Hearing responses were evaluated via maximum word recognition scores (WRSs) and pure-tone audiogram (PTA) testing. Improvement or deterioration in hearing were defined as a change of at least 10% in WRS. For evaluation of PTA, improvement or deterioration of hearing were defined as a change of at least 10 dB from 1 to 3 kHz.

Magnetic resonance imaging

Experimental design

Design type	Functional MRI was not used in this study.
Design specifications	Functional MRI was not used in this study.
Behavioral performance measures	Measure the size of tumor<br>Mean and standard deviation across subjects by NF2

Acquisition

Imaging type(s)	Structural image using gadolinium-enhanced MRI and CT perfusion.
Field strength	3.0T
Sequence & imaging parameters	Gadolinium-enhanced MRI (Tumor volume): FOV 256mm matrix 256x256 slice thickness 1mm T1WI cube (3D FSE) TR/TE 400/12.5, GE discovery 750<br>CT perfusion (Tumor blood volume): CT perfusion was obtained with area detector CT (Aquillion one;Canon medical system) and analyzed with the application on the CT.
Area of acquisition	a whole brain scan

Diffusion MRI ☐ Used ☒ Not used

Preprocessing

Preprocessing software	Gadolinium-enhanced MRI: FOV 256mm matrix 256x256 slice thickness 1mm T1WI cube (3D FSE) TR/TE 400/12.5, GE discovery 750<br>CT perfusion: area detector CT (Aquillion one;Canon medical system) and analyzed with the application on the CT.
Normalization	Normalization was not needed for this study.
Normalization template	Normalization was not needed for this study.
Noise and artifact removal	CT images were reconstructed with hybrid iterative reconstruction (AIDR 3D: Adaptive Iterative Dose Reduction 3D)
Volume censoring	An ROI was measured by tracing a line freehand around the tumor margins by using the cursor and mouse.

Statistical modeling & inference

Model type and settings	Univariate, Linear model
Effect(s) tested	Comparison between NF2 patients and non-NF2 patients. Comparison between pre- and post- vaccination.

Specify type of analysis: ☐ Whole brain ☐ ROI-based ☒ Both

Anatomical location(s)	Computed tomography (CT) and magnetic resonance imaging (MRI) were used to evaluate clinical responses. Tumor size was volumetrically measured via gadolinium-enhanced imaging. Tumor blood volume (TBV) was measured via CT perfusion as a ratio of the relative values to the lesion of interest in normal-appearing white matter.
Statistic type for inference<br>(See Eklund et al. 2016)	An ROI was measured by tracing a line freehand around the tumor margins by using the cursor and mouse.
Correction	Not applicable

Models & analysis

n/a	Involved in the study
☒	☐ Functional and/or effective connectivity
☐	☒ Graph analysis
☒	☐ Multivariate modeling or predictive analysis

Graph analysis	Student's t-test was used to compare Foxp3-positive cell counts, microvessel density (MVD), vessel diameter, and the expression of VEGF-A, VEGFR1, and VEGFR2 analyzed via qPCR in NF2 and non-NF2 patients. The paired t-test was used to assess radiographic changes post-vaccination. In the present study, treatment response was statistically assessed by comparing clinical course after vaccination with that before vaccination (as control) in each case. A P-value of <0.05 was considered statistically significant. Because we conducted only one statistical test pertaining to our conclusion, we did not apply correction for multiple comparisons.
